# Supplementary material for: DNAzyme Sensor for the Detection of Ca2+ Using Resistive Pulse Sensing
Source: Sensors (Basel). 2020 Oct 17;20(20):5877. doi: 10.3390/s20205877 (PMC7589696; doi:10.3390/s20205877)
Supplement: Supplementary file 1 [file sensors-20-05877-s001.pdf]

Supplementary Information

DNAzyme Sensor for the Detection of Ca^{2+} using Resistive Pulse Sensing

I. Heaton, M. Platt[1*]

1. Department of Chemistry, Loughborough University, Loughborough, Leicestershire. LE11 3TU, UK.

*m.platt@lboro.ac.uk

Table of contents

1. Difference in size between nanocarriers functionalised with just the substrate strand and those functionalised with the DNAzyme complex...................... S2
2. Comparison of different methods of cooling the DNAzyme complex post heating.. S3
3. Comparison of CPC200, 15T DNA and DNAzyme particle speed with and without Ca^{2+} present... S4
4. DNAzyme nanocarriers in the presence of 3 to 3000 µM Ca^{2+}................... S5
5. Differences between different batches of nanocarriers with and without 3 µM Ca^{2+} present.. S6
6. Frequency vs blockade magnitudes in the presence of Ca^{2+}...................... S7
7. DNAzyme functionalised nanocarriers incubated with 5 mM Mg^{2+} from 0 to 6 hours.. S8

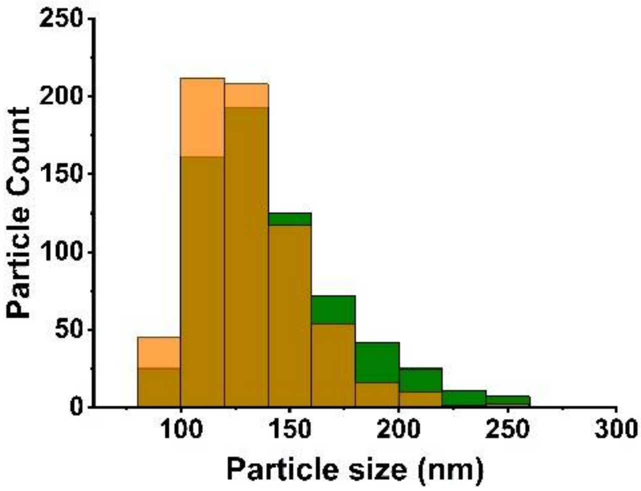

Figure S1: No change in particle size of substrate strand, orange, on the particles vs. DNAzyme complex, green, on the particle. Size compared to CPC200s, known size 210 nm, ran under the same conditions on the same day as the DNA samples, over 600 particles were counted.

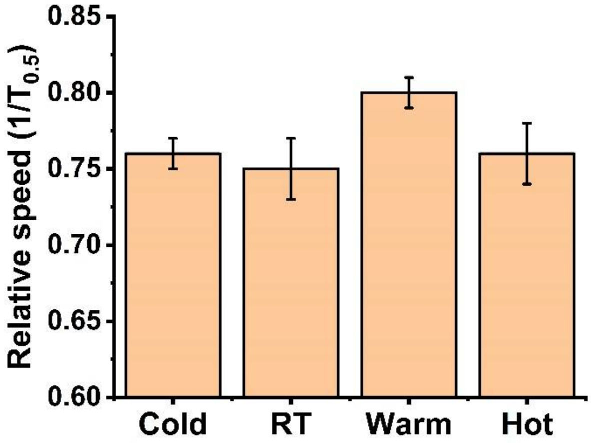

Figure S2: Varying the cooling process of the DNAzyme by placing it in the fridge, ~ 6 °C (cold), at room temperature, ~ 18 °C (RT), in a warm place, ~ 25 °C (warm) and left cooling in the dry bath, <70 °C (hot). Relative speed normalised to CPC calibration beads ran on the same day under the same conditions. Samples were ran in triplicate, more than 200 particles were measured each time. Error bars are one standard deviation from the mean.

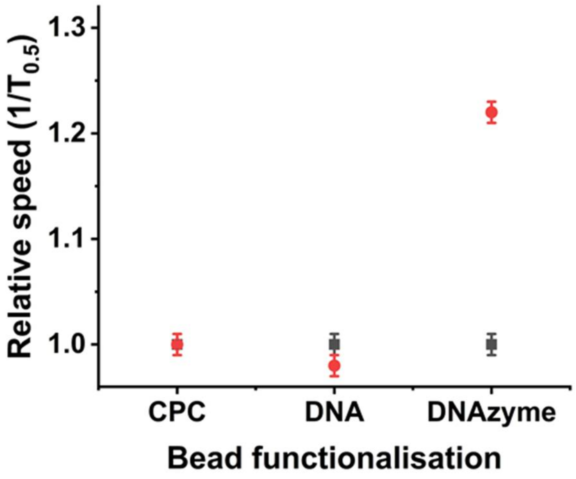

Figure S3: Comparison of different controls ran with (red) and without (black) 3 µM Ca^{2+} present, incubated together for 30 mins, CPC200s, 15T ssDNA strand and DNAzyme complex. Relative speeds taken as $1/T_{0.5}$ normalised to the blank particles without Ca^{2+}. Ran under the same conditions on the same day, each sample was ran in triplicate and more than 200 particles were measured each time.

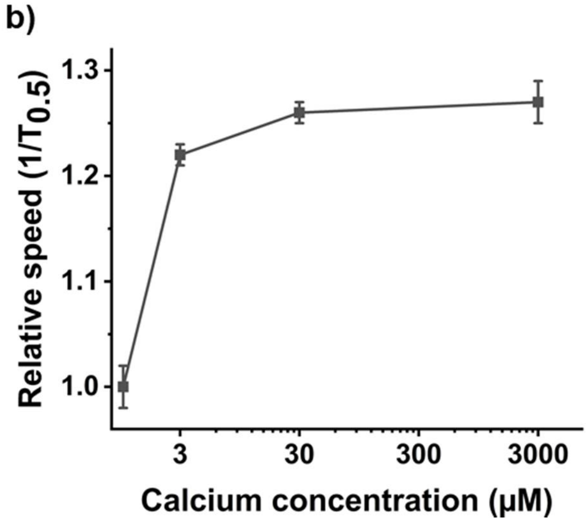

Figure S4: Relative particle speeds normalised to blank DNAzyme functionalised particles, vs. varying amounts of Ca^{2+} ions added from 3 – 3000 μM. Samples were ran in triplicate, more than 200 particles were measured each time. Error bars are one standard deviation from the mean.

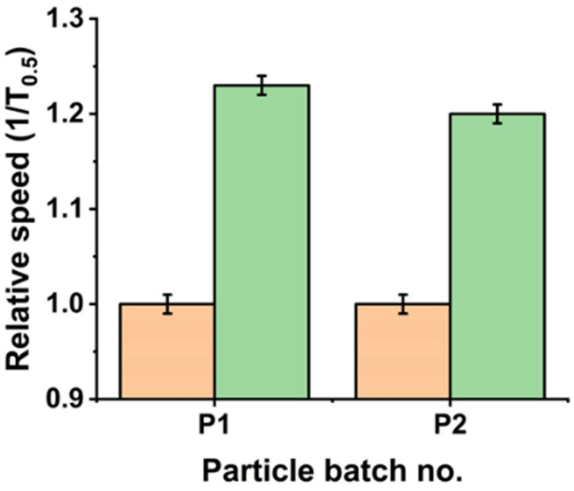

Figure S5: Comparison of two different batches of streptavidin particles, different blank particles ran with (green) and without (orange) 3 μM Ca^{2+} present. Relative speeds taken as $1/T_{0.5}$ normalised to the blank DNAzyme functionalised particles, without Ca^{2+} present, ran under the same conditions on the same pore and day. Each sample was ran in triplicate and more than 200 particles were measured each time.

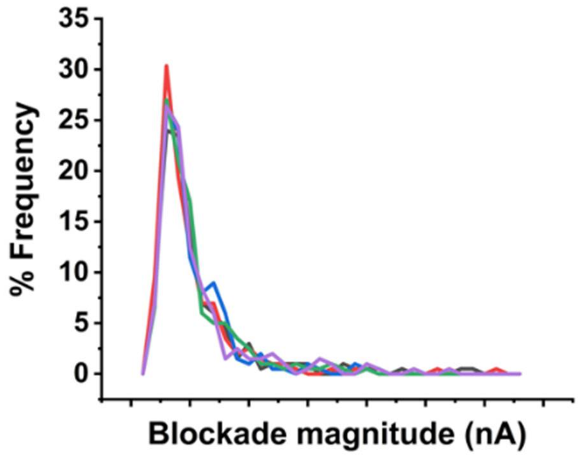

Figure S6: No change in the blockade magnitude indicating there is no particle aggregation, when Ca^{2+} present in concentrations from 1 – 9 μM.

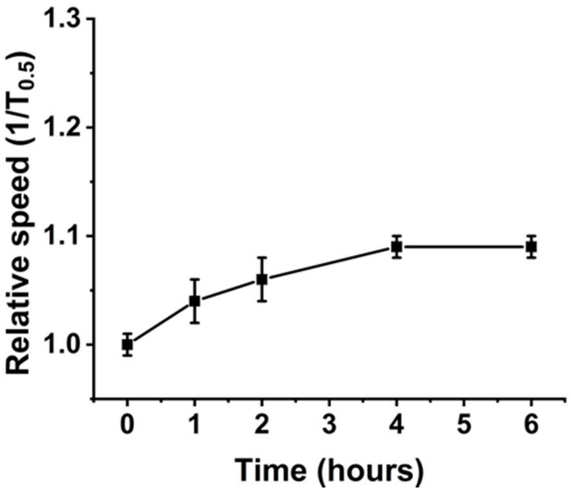

Figure S7: DNAzyme functionalised beads incubated with 5 mM Mg^{2+} over a period of 6 hours, samples taken from the stock hourly. Relative speeds taken as $1/T_{0.5}$ normalised to the blank particles without Mg^{2+}. Ran under the same conditions on the same day, each sample was ran in triplicate and more than 200 particles were measured each time.
